# Antimicrobial effects of mustard oil-containing plants against oral pathogens: an in vitro study

**DOI:** 10.1186/s12906-020-02953-0

**Published:** 2020-05-24

**Authors:** Vanessa Eichel, Anne Schüller, Klaus Biehler, Ali Al-Ahmad, Uwe Frank

**Affiliations:** 1 Center for Infectious Diseases, Heidelberg University Hospital, Im Neuenheimer Feld 324, 69120 Heidelberg, Germany; 2 Institute for Infection Prevention and Hospital Epidemiology, University of Freiburg, Breisacher Straße 115 B, 79106 Freiburg, Germany; 3 Department of Operative Dentistry and Periodontology, Freiburg University Hospital, Hugstetterstrasse 55, 79106 Freiburg, Germany

**Keywords:** Periodontitis, Isothiocyanates, Mustard oils, Endocarditis prophylaxis, Horseradish, Nasturtium

## Abstract

**Background:**

The present study examines the antimicrobial activity of nasturtium herb (*Tropaeoli maji herba*) and horseradish root (*Armoraciae rusticanae radix*) against clinically important oral bacterial pathogens involved in periodontitis, gingivitis, pulpitis, implantitis and other infectious diseases.

**Methods:**

A total of 15 oral pathogens, including members of the genera *Campylobacter, Fusobacterium, Prevotella, Parvimonas, Porphyromonas, Tanerella, Veillonella, and HACEK* organisms, were exposed to [1] a combination of herbal nasturtium and horseradish using a standardized gas test and [2] a mixture of synthetic Isothiocyantes (ITCs) using an agardilution test. Headspace gas chromatography mass spectrometry was employed to quantify the amount of allyl-, benzyl-, and 2- phenyl- ethyl-ITC.

**Results:**

With exception of *Veillonella parvula,* all tested species were highly susceptible to herbal nasturtium and horseradish in the gas test with minimal inhibitory concentrations (MICs) between 50/20 mg and 200/80 mg and to synthetic ITCs in the agardilution with MICs between 0.0025 and 0.08 mg ITC/mL, respectively. Minimal bactericidal concentrations extended from 0.005 mg ITC/mL to 0.34 mg ITC/mL.

**Conclusions:**

ITCs may be considered an interesting alternative to antibiotics for prevention and treatment of oropharyngeal infections, periodontitis and related diseases. Furthermore, the suitability of ITCs for endocarditis prophylaxis in dental procedures might be worth further investigation.

## Background

With increasing spread of antibiotic-resistant pathogens, and better understanding of the effects of antibiotics on the microbiota, alternatives to antibiotics must be considered for therapy and prevention.

The discovery in 1928 of penicillin by Alexander Fleming heralded the golden era of antibiotics, which, lasting until the late 1960s, saw the development of different novel antibiotic classes [1, 2]. Many bacterial pathogens, however, developed resistance to most of these antibiotics, and the paucity in the development of new antibiotics from the 1970s to the present day threatens a return to the preantibiotic era [1–4]. Additionally, resistance to disinfectants such as chlorhexidine digluconate may correlate with antibiotic resistance [5–7]. Due to the correlation observed between resistance against disinfectants and antibiotics, widespread use of disinfectants should be reassessed [6]. Considering the aforementioned points, there is substantial need for alternative treatment methods to control oral infections.

Nasturtium (*Tropaeolum majus* L*.*, TR) herb and horseradish (*Armoracia rusticana* P.Gaertn., B.Mey. & Scherb., AR) root release high amounts of isothiocyanates (ITCs) when glucosinolates - the inactive prodrugs of ITCs found naturally in *Brassica* plants - are hydrolysed by myrosinases, which are also present in *Brassica* plants [8, 9]. Several in-vitro studies have demonstrated that herbal ITCs display antimicrobial effects against a variety of bacteria including multidrug-resistant (MDR) bacteria, such as methicillin-resistant *Staphylococcus aureus,* vancomycin-resistant Enterococci*,* MDR *Escherichia coli*, penicillin-resistant *Streptococcus pneumoniae*, biofilm-producing *Pseudomonas aeruginosa* and also against viruses [10–14]. Clinical studies have demonstrated the non-inferiority of TR/AR compared to standard antibiotics in upper respiratory tract infections such as acute sinusitis and bronchitis treatment [15, 16] and their efficacy in prophylaxis of both respiratory and urinary tract infections [17, 18].

The objective of this in-vitro study was to assess the antimicrobial effects of TR/AR on clinically important oral pathogens. A total of 15 bacterial strains, (*Campylobacter concisus, Campylobacter rectus, Fusobacterium naviforme, Fusobacterium nucleatum, Parvimonas micra, Prevotella baroniae, Prevotella intermedia, Porphyromonas gingivalis, Tannerella forsythia, Veillonella parvula),* including 5 HACEK organisms (*Haemophilus aphrophilus, Aggregatibacter actinomycetemcomitans, Cardiobacterium hominis*, *Eikenella corrodens*, *Kingella kingae),* involved in periodontitis and other diseases and one reference strain (*Clostridium perfringens*) were assessed. Table 1 gives an overview of the diseases these strains may cause.

**Table 1:** Diseases caused by the bacterial strains tested (adapted from (Lamont and Jenkinson 2010))

Disease	Involved species
Gingivitis	*Fusobacterium* spp.*, Prevotella* spp.*, Campylobacter* spp.
Periodontitis	*Aggregatibacter actinomycetemcomitans, Campylobacter* spp.*, Eikenella corrodens, Fusobacterium* spp.*, Porphyromonas gingivalis, Prevotella* spp.*, Tannerella forsythia, Veillonella parvula*
Implantitis	*Porphyromonas gingivalis, Prevotella* spp.
Pulpitis	*Fusobacterium* spp.*, Parvimonas micra, Porphyromonas ginigvalis*
Halitosis	*Fusobacterium* spp.*, Porphyromonas gingivalis, Prevotella* spp.
Pharyngitis	*Haemophilus aphrophilus*
Tonsillitis	*Haemophilus aphrophilus*
Meningitis	*Veillonella parvula*
Endocarditis	*Haemophilus aphrophilus, Aggregatibacter actinomycetemcomitans, Cardiobacterium hominis, Eikenella corrodens, Kingella kingae*

## Methods

### Headspace gas chromatography mass spectrometry

To identify the amount of allyl–ITC, benzyl–ITC, and 2-phenyl-ethyl–ITC in TR and AR, headspace gas chromatography mass spectrometry (GC-MS) was employed. For calibration 0.1, 0.5, 1, 2.5, or 5 μL of a ITC stock solution (ITC:Methanol = 1:100) was added to 500 μL H_2_0 in a 10 mL glass vial. After 30 min shaking at 60 °C 500 μL gas were analyzed using a GC-MS-QP2010S (Shimadzu) equipped with a 30 m × 0.32 mm HP-VOC capillary column. The flow rate of the carrier gas helium was 1 mL/min. The column temperature was programmed from 60 °C to 220 °C at a rate of 10 °C/min. The temperatures of the injector and detector were set to 200 °C and 280 °C, respectively. The experiments were carried out with electron impact ionization (EI) mode at electron energy of 70 eV. The degradation products were identified by matching the recorded mass spectra with the NIST 107 mass spectrum library of the GC-MS data system. Mass-to-charge ratios for benzyl-ITC were 149 m/z and 91 m/z, for allyl–ITC 99 m/z and 41 m/z, and for 2-phenyl-ethyl–ITC 163 m/z and 91 m/z, respectively. For testing TR and AR, 1 mg of the dried plant was activated with 500 μL H_2_0 and ITCs amounts were measured in the same way as the calibration stock.

### Cultivation of bacterial strains

The bacterial strains were chosen from the collection of the microbiological institute of the university hospital of Freiburg, Germany. They were cultivated under different conditions corresponding to their special needs. Yeast-cysteine-blood (YCB) agar plates containing 5% sheep blood were prepared. The anaerobes *Aggregatibacter actinomycetemcomitans, Campylobacter concisus, Campylobacter rectus, Clostridium perfringens, Parvimonas micra, Prevotella baroniae, Prevotella intermedia, Porphyromonas gingivalis, Tannerella forsythia,* and *Veillonella parvula* were cultured on the agar plates in Anaerocult pots (Becton Dickinson and Merck) in which the anaerobic conditions were monitored by Dry Anaerobic Indicator Strips (Becton Dickinson). The microaerophilic strains, *Fusobacterium naviforme and nucleatum, Haemophilus aphrophilus, Kingella kingae, Eikenella corrodens* and *Cardiobacterium hominis* grew at 5% CO_2._ The plates were incubated at 36.5 °C, and 32.5 °C for *Prevotella intermedia* and *Prevotella baronie.*

### Preparation of bacterial stock solution

To achieve quantitative results, a defined amount of bacteria should be used for sensitivity tests. The bacterial stock solutions were prepared by harvesting bacterial colonies from the plates with sterile cotton swabs and suspending them in 1 mL PBS (Dulbecco Biochrom). The turbidity of the suspensions was adjusted to McFarland standard 0.5, which corresponds to a concentration of approximately 10^8^ cells/mL. The solution was diluted with PBS to a final concentration of 10^4^ cells/mL.

### Phytotherapeutic drug susceptibility testing with gas test

**Fig. 1:**
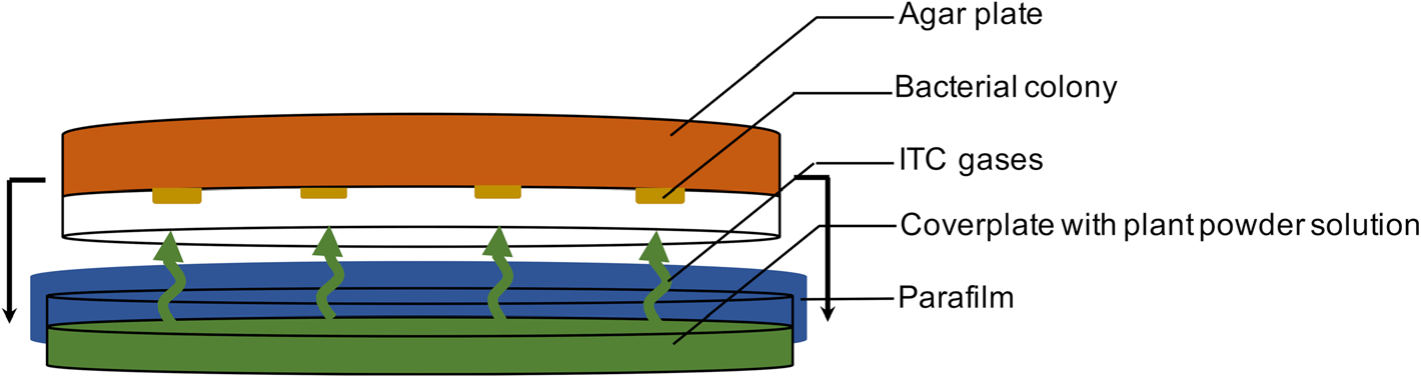
Opened apparatus for gas test experiments

The antimicrobial effects of the herbal drugs were assessed using a modified gas test (Fig. 1). Cover plates were filled with the native substances TR herb powder (18,834, supplier Martin Bauer) and AR root powder (17,604, supplier Peter) at a ratio of 2.5:1 (REPHA GmbH, Langenhagen, Germany). Amounts of TR/AR ranged from 12.5/5 mg, which corresponds to 1/16 of the commercially available tablet “Angocin®” and to 400/160 mg, which corresponds to 2 tablets. A negative control with an empty cover plate was added to each series. The bacterial stock solutions were spread on YCB agar plates and the drugs were activated by stirring in 2 mL PBS. The plates were assembled, closed with parafilm, and incubated at 32.5 °C and checked for bacterial growth after 48 and 72 h, respectively, by counting colony forming units (CFU). Minimal inhibitory concentrations (MIC) were defined as the lowest concentration of TR/AR to prevent visible bacterial growth. Every strain was tested at least twice with this assay. MIC values are mean values.

### ITC susceptibility testing with agardilution

To verify the effects observed in the gas test, synthetic ITCs were used to perform agar dilution tests. To reflect the proportions of active agents in Angocin®, a mixture of 38% allyl-ITC (Merck), 50% benzyl-ITC (Sigma-Aldrich), and 12% 2-phenyl-ethyl-ITC (Fluka) was prepared. 1% (v/v) polysorbate 80 (Merck Schuchardt) served as a solvent to dilute the lipophilic mixture. The ITC/polysorbate mixture was diluted nine times 1:1 to prepare the desired concentrations. Series of YCB agar plates with ITC concentrations from 0.0025 mg/mL to 0.34 mg/mL were poured by adding 2 mL ITC/polysorbate mixture to 18 mL liquid YCB agar. The bacterial stock solution with a concentration of 10^4^ cells/mL was inoculated using a multipoint-inoculator (Mast). As a negative control, 2 mL polysorbate without ITC was added to the YCB agar. The inoculated agar plates were incubated at 32.5 °C up to 5 days, depending on the particular bacterial growth rate. Anaerobes were cultivated in Anaerocult pots, microaerophilic bacteria at 5% CO_2_. The plates were checked for bacterial growth after 48 and 72 h, respectively. Each strain was tested at least twice with this assay. The minimal bactericidal concentration (MBC) is the lowest ITC concentration required to kill the bacterium. For determination of the MBC values, inoculated areas which showed no visible bacterial growth after 48 h incubation were transferred with a sterile swab to an ITC-free YBC agar plate, and checked for bacterial growth after 48 and 72 h, respectively. Each strain was tested at least twice with this assay. MIC and MBC values are mean values.

## Results

### Horseradish and Nasturtium powder release high amounts of Isothiocyanates

**Fig. 2:**
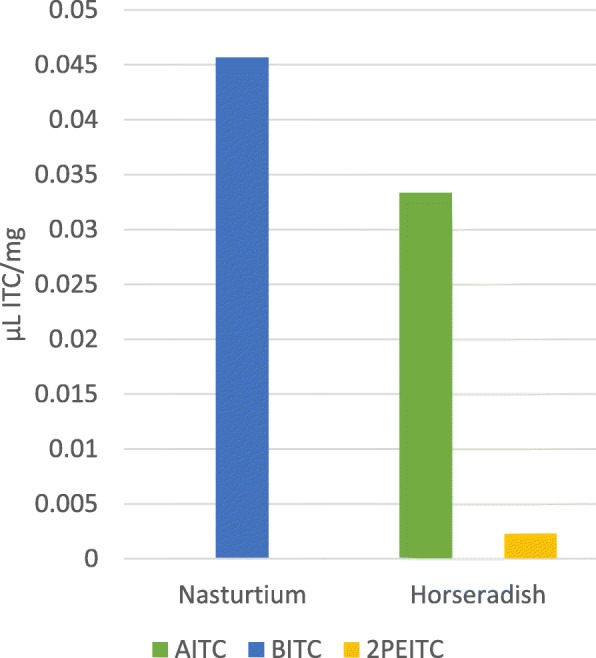
GC-MS defined ITC amounts in Nasturtium and Horseradish; *AITC*: allyl– ITC; *BITC*: benzyl– ITC, *2PEITC*: 2- phenyl- ethyl– ITC; *n* = 3

With headspace GC-MS we measured considerable amounts of ITCs in the gases, when *Tropaeolum majus* and *Armoraciae rusticanae* powder was activated with water (Fig. 2). While TR mainly contained benzyl–ITC (0.046 ± 0.001 μL/mg), AR released huge amounts of allyl–ITC (0.033 ± 0.001 μL/mg), and 2-phenyl-ethyl–ITC (0.0023 ± 0.0005 μL/mg).

### Phytotherapeutic drugs inhibit bacterial growth

**Fig. 3:**
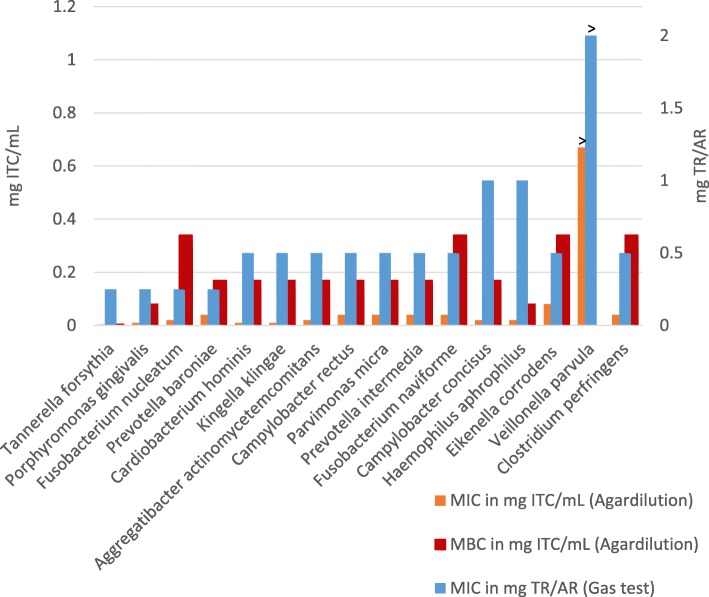
*blue:* MIC values of TR/AR in gas tests (right scale); *orange, red:* MIC and MBC values of synthetic isothiocyanates in agardilution tests (left scale); >: maximum test concentration reached; *n* = 2

All species were highly susceptible to herbal TR/AR in the gas test and to synthetic ITC in the agar dilution test, except for *Veillonella parvula.* MIC values were determined between 50/20 mg and 200/80 mg TR/AR, and 0.0025 and 0.08 mg ITC/mL, as shown in Fig. 3. The highest susceptibility was shown for *Tannerella forsythia* in both, the gas test and the agardilution. However, in the gas test growth of *Porphyromonas gingivalis, Fusobacterium nucleatum,* and *Prevotella baroniae* was also inhibited at 50/20 mg TR/AR. The agardilution experiments revealed that after *Tannerella Porphyromonas ginigvalis, Cardiobacterium hominis,* and *Kingella klingae* were next susceptible to ITCs. MBCs extended from 0.005 mg ITC/mL for *Tannerella forsythia* to 0.34 mg ITC/mL for *Fusobacterium naviforme, Fusobacterium nucleatum,* and *Eikenella corrodens*. Growth of *Veillonella parvula* was not influenced by TR/AR or synthetic ITCs at the concentrations tested. Negative control plates without antibiotic agents showed normal bacterial growth.

## Discussion

### High antimicrobial effects

The antimicrobial effects of *Tropaeolum majus* L. (TR) and *Armoracia rusticana* P.Gaertn., B.Mey. & Scherb (AR) against oral pathogenic bacteria have not been studied sufficiently, so far. Hence, the aim of our study was to determine the susceptibilities of clinically important oral pathogens and to show that TR and AR are feasible for the usage in antimicrobial therapy in patients.

In addition to the frequently used standard MIC-Methods a modified gas test set up was used to evaluate the antimicrobial effects of the dried plant powder [19, 20].

Prior to testing the antimicrobial activity, the active substances of TR and AR were analyzed in detail using headspace gas chromatography mass spectrometry (GC-MS). We found chemically different ITCs in the two plants, which favors the use of a combination of ITC-containing plants. For our susceptibility tests, we therefore used a mixture of TR and AR at a proportion of 2.5:1 and a combination of synthetically produced ITCs with matches the proportions of ITCs in the plants.

With the exception of *Veillonella parvula,* all tested species were highly susceptible to herbal TR/AR in the gas test and synthetic ITCs in the agar dilution, with MICs ranging between 50/20 mg and 200/80 mg TR/AT, and 0.01 and 0.08 mg ITC/mL, respectively. *Tannerella forsythia, Porphyromonas gingivalis, Fusobacterium nucleatum* and *Prevotella baroniae* were found to be the most sensitive of the tested species, as 50/20 mg TR/AR, which equates to only 1/4 tablet of the commercially available drug (Angocin®), was enough to stop growth. MBC values were about 4 to 10 times higher than MIC values. Although the order of herbal MIC values does not exactly correlate with the sequence of synthetic ITC MIC and MBC values, all determined concentrations could easily be reached in the oral cavity by topical application as mouthwash, gel, chip, or, in combination with fluoride, as toothpaste.

Our GC-MS experiments demonstrated, that a mixture of herbal TR/AR powder can release a spectrum of ITCs in high amounts. Previous studies tested TR alone for its bactericidal effect on certain oral pathogens. However, the ITC solution was sucked in a paper disk and placed on the agar. The MBC values of the ITCs extracted from TR and the synthetic allyl- ITC were higher than in our experiments [21]. This could mean that the combination of TR and AR is more effective than TR alone.

Furthermore, *Salvadora persica,* sticks of which have been used as natural toothbrushes for centuries, were found to contain high amounts of benzyl-ITC and show considerable antimicrobial effects on *Aggregatibacter actinomycetemcomitans* and *Porphyromonas gingivalis* [22]. This is consistent with the results presented in this study for ITCs.

### Why phytotherapeutic drugs?

Although TR and AR are cultivated and have been used for hundreds of years, relevant bacterial resistances to ITCs have not as yet been reported. The safety of systemic TR/AR administration up to 1200/480 mg daily was demonstrated in a clinical trial [17]. Adverse side effects were significantly lower in a TR/AR group than an antibiotic group [16]. Negative effects on the gut microbiota were not observed. Moreover, the treatment costs with TR/AR are substantially lower than with antibiotic prescriptions. Additionally, chlorhexidine, which has been considered the gold standard in dental plaque control [23], is also cytotoxic, as reported for human gingival fibroblasts, osteosarcoma cells and osteoblasts [24, 25]. Moreover, human saliva can to some extent inactivate the antibacterial effects of chlorhexidine against some oral bacteria, inducing selective processes in the bacterial populations of human saliva [26]. Furthermore, a correlation of resistance towards chlorhexidine and different medically relevant antibiotics cannot be excluded due to the similar mechanisms of resistance which include multidrug efflux pumps and cell membrane changes as reported in an own review of the literature [27]. Another frequently used oral health product is Listerine® [28]. Although there is accumulating evidence that Listerine® is effective in improving oral health, the absence of systematic toxicological studies means that an accurate safety assessment cannot be made [29]. Hence, new natural antibacterial compounds such as ITCs from plants could be promising components for dental oral care. However, the direct comparison of ITCs effects on oral pathogens with standard antibiotics or chlorhexidine is still pending, which must be acknowledged as a limitation of our study approach.

### Clinical benefit

Out of the tested species, *Aggregatibacter actinomycetemcomitans, Campylobacter rectus, Eikenella corrodens, Fusobacterium nucleatum, Porphyromonas gingivalis, Prevotella intermedia, Tanerella forsythia,* and *Veillonella parvula* are highly associated with periodontitis [30, 31]. With the exception of *Veillonella parvula,* all these pathogens were found to be highly susceptible to ITCs. The topical use of herbal TR/AR, e.g. as antiseptic mouthwash, gel or chip, should be considered, but also systemic administration, since the compliance to phytotherapy is usually good, and spread of antibiotic resistance could be avoided. Activity exhibited by ITCs against biofilms was demonstrated by the example of *Pseudomonas aeruginosa* [12]. The effects against the diverse array of oral bacteria tested in the present study suggest an anti-biofilm effect of ITCs. Such potential should be examined in future studies to clarify inhibition of formation or degradation of already formed oral biofilm.

Endocarditis prophylaxis for dental procedures should predominantly cover *Staphylococci*, *Streptococci, Enterococci, and Candida* spp.*,* but also incidental pathogens such as HACEK organisms [32]. Our in vitro*-*study demonstrated that HACEK organisms are highly susceptible to TR/AR. These results support and expand our previous findings of the antibacterial effect of mustard oil-containing plants against the predominant endocarditis relevant oral bacteria [10].

## Conclusions

This study showed that different components of mustard oil-containing plants have a high antimicrobial activity against various oral bacteria. The presented results suggest a high potential activity against oral biofilm formation which should be tested in vivo in future clinical studies to evaluate their beneficial protective effects to prevent oral diseases such as caries, periodontitis and periimplantitis.

## Supplementary information

**Additional file 1: Table S1.** Exposed species and its corresponding MIC values of nasturtium herb and horseradish root in gas tests, as well as MIC and MBC values of synthetic isothiocyanates in agardilution tests; *n.d*: not defined.

## Data Availability

The datasets used and/or analysed during the current study are available from the corresponding author on reasonable request.

## Abbreviations

AR: *Armoracia rusticana* P.Gaertn., B.Mey. & Scherb
CFU: Colony forming units
ITC: Isothiocyanate
MBC: Minimal bactericidal concentration
MDR: Multidrug-resistant
MIC: Minimal inhibitory concentration
TR: *Tropaeolum majus* L.
YCB: Yeast-cysteine-blood
