# Supplementary material for: Antimicrobial effects of mustard oil-containing plants against oral pathogens: an in vitro study
Source: BMC Complement Med Ther. 2020 May 24;20:156. doi: 10.1186/s12906-020-02953-0 (PMC7247255; doi:10.1186/s12906-020-02953-0)
Supplement: Supplementary file 1 — Additional file 1: Table S1. Exposed species and its corresponding MIC values of nasturtium herb and horseradish root in gas tests, as well as MIC and MBC values of synthetic isothiocyanates in agardilution tests; n.d: not defined. [file 12906_2020_2953_MOESM1_ESM.docx]

**Table S1**

Exposed species and its corresponding MIC values of nasturtium herb and horseradish root in gas tests, as well as MIC and MBC values of synthetic isothiocyanates in agardilution tests; *n.d*: not defined

|  | **Gas test** | **Agardilution** | **Agardilution** |
| --- | --- | --- | --- |
| **Species** | **MIC in mg TR/AR (in tablets Angocin)** | **MIC in mg ITC/mL** | **MBC in mg ITC/mL** |
| *Tannerella forsythia*  *Porphyromonas gingivalis* | 50/20 (0.25)  50/20 (0.25) | 0.0025  0.01 | 0.005  0.08 |
| *Fusobacterium nucleatum* | 50/20 (0.25) | 0.02 | 0.34 |
| *Prevotella baroniae* | 50/20 (0.25) | 0.04 | 0.17 |
| *Cardiobacterium hominis* | 100/40 (0.5) | 0.01 | 0.17 |
| *Kingella klingae* | 100/40 (0.5) | 0.01 | 0.17 |
| *Aggregatibacter actinomycetemcomitans* | 100/40 (0.5) | 0.02 | 0.17 |
| *Campylobacter rectus* | 100/40 (0.5) | 0.04 | 0.17 |
| *Parvimonas micra* | 100/40 (0.5) | 0.04 | 0.17 |
| *Prevotella intermedia* | 100/40 (0.5) | 0.04 | 0.17 |
| *Fusobacterium naviforme* | 100/40 (0.5) | 0.04 | 0.34 |
| *Campylobacter concisus* | 200/80 (1) | 0.02 | 0.17 |
| *Haemophilus aphrophilus* | 200/80 (1) | 0.02 | 0.08 |
| *Eikenella corrodens* | 200/80 (0,5) | 0.08 | 0.34 |
| *Veillonella parvula* | > 400/160 (> 2) | > 0.67 | n.d. |
| *Clostridium perfringens* | 200/80 (0,5) | 0.04 | 0.34 |
